# Regulation of hERG and hEAG Channels by Src and by SHP-1 Tyrosine Phosphatase via an ITIM Region in the Cyclic Nucleotide Binding Domain

**DOI:** 10.1371/journal.pone.0090024

**Published:** 2014-02-28

**Authors:** Lyanne C. Schlichter, Jiahua Jiang, John Wang, Evan W. Newell, Florence W. L. Tsui, Doris Lam

**Affiliations:** 1 Genes and Development Division, Toronto Western Research Institute, University Health Network, Toronto, Ontario, Canada; 2 Department of Physiology University of Toronto, Toronto, Ontario, Canada; 3 Department of Immunology, University of Toronto, Toronto, Ontario, Canada; SUNY College of Nanoscale Science and Engineering, United States of America

## Abstract

Members of the EAG K^+^ channel superfamily (EAG/Kv10.x, ERG/Kv11.x, ELK/Kv12.x subfamilies) are expressed in many cells and tissues. In particular, two prototypes, EAG1/Kv10.1/*KCNH1* and ERG1/Kv11.1/*KCNH2* contribute to both normal and pathological functions. Proliferation of numerous cancer cells depends on hEAG1, and in some cases, hERG. hERG is best known for contributing to the cardiac action potential, and for numerous channel mutations that underlie ‘long-QT syndrome’. Many cells, particularly cancer cells, express Src-family tyrosine kinases and SHP tyrosine phosphatases; and an imbalance in tyrosine phosphorylation can lead to malignancies, autoimmune diseases, and inflammatory disorders. Ion channel contributions to cell functions are governed, to a large degree, by post-translational modulation, especially phosphorylation. However, almost nothing is known about roles of specific tyrosine kinases and phosphatases in regulating K^+^ channels in the EAG superfamily. First, we show that tyrosine kinase inhibitor, PP1, and the selective Src inhibitory peptide, Src40-58, reduce the hERG current amplitude, without altering its voltage dependence or kinetics. PP1 similarly reduces the hEAG1 current. Surprisingly, an ‘immuno-receptor tyrosine inhibitory motif’ (ITIM) is present within the cyclic nucleotide binding domain of all EAG-superfamily members, and is conserved in the human, rat and mouse sequences. When tyrosine phosphorylated, this ITIM directly bound to and activated SHP-1 tyrosine phosphatase (PTP-1C/PTPN6/HCP); the first report that a portion of an ion channel is a binding site and activator of a tyrosine phosphatase. Both hERG and hEAG1 currents were decreased by applying active recombinant SHP-1, and increased by the inhibitory substrate-trapping SHP-1 mutant. Thus, hERG and hEAG1 currents are regulated by activated SHP-1, in a manner opposite to their regulation by Src. Given the widespread distribution of these channels, Src and SHP-1, this work has broad implications in cell signaling that controls survival, proliferation, differentiation, and other ERG1 and EAG1 functions in many cell types.

## Introduction

The ‘*ether-a-go-go*’ gene family, which encodes alpha subunits of six-transmembrane domain voltage-gated K^+^ channels, has been divided into three sub-families: *EAG*, EAG-related gene (ERG) and EAG-like (*ELK*) K^+^ channels. The two prototypes, EAG1/Kv10.1/*KCNH1* and ERG1/Kv11.1/*KCNH2*, normally have a restricted tissue expression, but are mutated or aberrantly expressed in several disease states. For instance, EAG1 is normally expressed in the brain and peripheral ganglia [1]–[3] and is transiently expressed in undifferentiated myoblasts [4] but it is aberrantly expressed in many tumor cells, where it might be a useful cancer marker and even a potential therapeutic target [5]–[7]. ERG1 has garnered enormous attention because many of its naturally occurring mutations, and numerous medications inhibit channel function and lead to life-threatening cardiac arrhythmias [8]–[9]. ERG1 is normally expressed in cardiac and vascular smooth muscle, the brain, thymus and adrenal gland [10], but its aberrant expression in several malignant cell types has aroused interest in its contributions to cancer [11]–[15]. We provided the first evidence of selective up-regulation of human ERG1 (hERG) expression and function in primary leukemic cells and several hematopoietic cell lines, but not in proliferating noncancerous lymphocytes [12], and a similar result was seen in cells from acute myeloid leukemia patients [15].

The contributions of ion channels depend first on channel expression and proper trafficking; after which, many channels are regulated by a balance between phosphorylation by various protein kinases and de-phosphorylation by phosphatases. Src, the prototypical member of the large Src-family of non-receptor tyrosine kinases, is activated downstream of numerous receptors, and present in many cells and tissues in which ERG1 or EAG1 are important, including immune cells [16], cancer cells [12], [15], [17] and cardiac and vascular smooth muscle [18], [19]. Immune cells are activated through membrane receptors that recruit Src-family kinases, and are negatively regulated by receptors that signal to protein tyrosine phosphatases (PTPs), and an improper balance of these pathways can lead to malignancies, autoimmune diseases and inflammatory disorders [20]–[22]. Thus, the link between cancer, EAG1 and ERG1 raises the intriguing possibility that contributions of these channels depend on tyrosine phosphorylation.

In 2002, we published the first studies demonstrating that tyrosine phosphorylation regulates a member of the EAG/ERG/ELK family of K^+^ channels [16], [23]. That is, endogenous ERG-like currents in the MLS-9 cell line were regulated by Src and by SHP-1 (PTP-1C/PTPN6/HCP), a SH2 (Src homology 2)-containing tyrosine phosphatase predominantly expressed in hematopoietic cells. While the native current was very similar to ERG1, we could not rule out the possibility that it was produced by heteromultimeric channels; e.g., ERG1 and ERG2 were both present. Given the importance of EAG and ERG channels to human pathology, it is crucial to address regulation of the identified human channels, hEAG1 and hERG. However, surprisingly little subsequent work has been published, with no information concerning SHP-1 regulation of these channels. The two papers showing tyrosine phosphorylation of cloned hERG and hEAG1 suggested intriguing differences between these two channels. A Src-family kinase inhibitor (PP2) and an EGFR kinase inhibitor (AG556) modulated hERG [24], but only the EGFR inhibitor regulated hEAG1 [25].

Here, we address the regulation of hERG and hEAG1 channels by SHP-1 PTP, and direct regulation by Src. First, we show that heterologously expressed hERG and hEAG1 channels are regulated by Src-family kinases, and specifically, by Src itself. Then, having noted that all EAG-family members contain an ITIM motif in their cytoplasmic C-terminus, we show that this motif is functional and activates SHP-1. SHP-1 then regulates both channels in a manner opposite to Src. An abstract outlining our preliminary results was published in the proceedings of the 2006 Biophysical Society meeting [26].

## Materials and Methods

### Cells and reagents

Human embryonic kidney (HEK293) cells stably expressing hERG (from Dr. Terrance Snutch, University of British Columbia, Vancouver, BC, Canada) were grown in Dulbecco's Modified Eagle's Medium (DMEM, Gibco, Invitrogen, Burlington ON), with 10% heat-inactivated fetal bovine serum (FBS), 100 units/mL penicillin and 100 µg/mL streptomycin. hEAG1 and pEGFP plasmids (2∶1) were transfected into HEK293 cells with Lipofectamine (5 µl/mL, 2 h) in Opti-MEM I. The MLS-9 cell line was cultured in MEM with 5% horse serum, 5% FBS, and 50 µg/mL gentamycin [16], [23], [27]–[28]. Media and reagents were from Gibco, Invitrogen (Burlington, ON, Canada), unless otherwise stated. The Src-family kinase inhibitor, PP1 (Enzo Scientific, Farmingdale, NY, USA) was made in DMSO and stored at −20°C. As before [16], recombinant Src40–58 peptide and a scrambled version (Src40–58s) were synthesized, prepared as concentrated stock solutions, stored at −80°C and thawed just before use. As before [23], [29], recombinant SHP-1 wt and SHP-1 C453S proteins were prepared, their sequences verified, expression of the fusion protein induced in liquid cultures, and the His-tagged proteins were purified using a Talon column (Clontech, Palo Alto, CA, USA).

### Patch-clamp electrophysiology

For recording hERG currents, stably transfected HEK293 cells were grown to ∼85% confluency, washed with PBS, re-suspended in 0.05% trypsin-EDTA solution, and transferred to DMEM. After dispersion at low density on glass coverslips, they were cultured for 18–20 h before use. hEAG1-transfected HEK293 cells on glass coverslips were cultured for 24–48 h in DMEM, and recordings were made from EGFP-positive cells. The hERG blocker, E-4031 (Alomone, Jerusalem, Israel), and the hEAG1 blocker, astemizole (Sigma, Oakville, ON, Canada), were used to confirm that the observed current was hERG or hEAG1, respectively.

Whole-cell currents were recorded with an Axopatch-200A amplifier and pCLAMP software (Axon Instruments, Foster City, CA, USA), and compensated on-line for series resistance and capacitance. Pipettes (3–4 MΩ) were filled with internal solution, containing (in mM): 125 KCl, 1 MgCl_2_, 1 CaCl_2_, 5 MgATP, 10 EGTA, and 10 HEPES (pH 7.2). The intracellular free Ca^2+^ concentration was 20 nM (calculated using WEB-MAXC Extended software; http://www.stanford.edu/~cpatton/webmaxcS.htm) and thus, no Ca^2+^ activated currents were present. Cells were superfused with bath solution (130 NaCl, 4.7 KCl, 2 CaCl_2_, 1 MgCl_2_, 10 HEPES, 5 glucose, pH 7.4) at room temperature (22±1°C). With both approaches used to study channel regulation, the current in each cell was normalized to its initial value. (i) Membrane-permeant PP1 was perfused into the bath after control currents were recorded, and separate control cells were exposed to 0.1% DMSO (the maximal solvent concentration used) to assess normal current rundown. (ii) Cell-impermeant Src peptides and recombinant SHP-1 proteins were added to the pipette solution and time-dependent changes were monitored as they diffused into the cells and exerted their effects.

### Biochemical assays

After finding an ITIM-like motif (LTYCDL: amino acids 825–830) in the C-terminus of hERG, we constructed a mutant channel (ΔITIM) lacking this sequence and several flanking amino acids (see below). To examine interactions between SHP-1 and hERG, HEK293 cells were transfected (using FUGENE6, Invitrogen) with wild-type or ΔITIM hERG constructs, incubated 48 h, treated with pervanadate (15 min, 37°C) and lysed. For co-immunoprecipitation, 250 µg of each lysate was incubated (45 min; room temperature (RT)) with 2 µg anti-SHP-1 antibody (sc-287, Santa Cruz Biotech., CA, USA), then incubated with protein-G beads (1 h, 4°C). The eluted antigen/antibody complex was run on SDS-PAGE, transferred to Immobilon, blocked with 5% BSA (1 h, 37°C), incubated (30 min, RT) with anti-hERG antibody (sc-20130, 1∶200; Santa Cruz), washed and incubated (30 min, RT) with the secondary antibody (HRP-conjugated anti-rabbit IgG, 1∶20,000; Jackson ImmunoResearch Labs, West Grove, PA, USA). After extensive washing, blots were incubated with SuperSignal West Femto Maximum Sensitivity Substrate (Pierce, Rockford, IL, USA) and visualized with a Bio-Rad Fluor-S MultiImager.

#### Pull-down assay

Tyrosine-phosphorylated (pY) or non-phosphorylated (Y) ITIM peptides plus flanking amino acids, ‘ITIM-hERG’ (see below) were synthesized, biotinylated and purified (United Biochem. Res., Seattle, WA, USA). Each peptide was incubated with recombinant SHP-1 C453S protein (1 h, 25°C) in 120 µL of lysis buffer (50 mM TRIS pH 8.0, 150 mM NaCl, 1% NP40, 1 mM EDTA, 20 µg/mL leupeptins, 10 µg/mL pepstatin A, 20 µg/mL antipain). Biotinylated ITIM-hERG/SHP-1 complexes were pulled down (1 h on ice) with avidin-immobilized agarose beads (NeutrAvidin, Pierce) in 1% BSA, followed by centrifugation. The NeutrAvidin beads were washed (6×) and proteins were eluted (boiled; 5 min) in 20 µL gel-loading buffer (65 mM Tris-HCl pH 6.8, 2% SDS, 2% dithiothreitol, 25% glycerol, 0.01% bromphenol blue, 1 mM Na_3_VO_4_). For Western analysis, proteins were separated on 10% polyacrylamide, electro-transferred to Immobilon-P (Millipore, Bedford, MA, USA) and blocked with 3% BSA in TBST (20 mM Tris-HCl, pH 8.0, 500 mM NaCl, 0.2% Tween 20). The membrane was incubated (45 min, 25°C) with anti-SHP-1 (1∶10,000; goat polyclonal custom-made by Sigma, affinity purified by Research Genetics, Huntsville, AL, USA) in TBST with 1% BSA. After washing (3×, TBST), the membrane was incubated (30 min, 25°C) with the secondary antibody (HRP-conjugated donkey anti-goat, 1∶50,000; Jackson Labs), washed (7×, TBST), and proteins were visualized by enhanced chemiluminescence (Amersham Biosciences, Buckinghamshire, UK) using a BioRad imager, as above. The same two biotinylated ITIM-hERG peptides were used to pull down endogenous SHP-1 from MLS-9 cell lysates following incubation with avidin-immobilized agarose beads (4°C, overnight) as above. Proteins were eluted, separated on 8% polyacrylamide, electrotransferred to PVDF membranes, and SHP-1 was identified by Western blotting.

#### Enzymatic assay

We used an enzymatic assay [30] to assess whether the SHP-1 enzyme is activated by the hERG channel ITIM motif. Purified, recombinant SHP-1 wt protein was incubated with varying concentrations of phosphorylated or non-phosphorylated ITIM-hERG peptide in 50 µl tyrosine phosphatase buffer (50 mM PIPES, pH 7.0, 150 mM NaCl, 2 mM EDTA, 2 mM DTT) containing the substrate, *p*-nitrophenyl phosphate (*p*NPP, Sigma). Conversion of *p*NPP to *p*-nitrophenol was detected at OD_410_ nm, and reactions were stopped with 450 µL of 0.2 M NaOH (after 3 h; 25°C). Product generation was linear up to 4 h. Results were analyzed by nonlinear least squares fitting of the rectangular hyperbolic form of the data using SigmaPlot (San Jose, CA, USA).

### Statistical analysis

In all graphs, the values are mean±SEM for the number of cells in parentheses, and *p*<0.05 was taken as statistically significant. A two-tailed paired or unpaired *t* test was used for single treatment comparisons. For multiple comparisons, ANOVA with post-hoc Bonferroni's test was used. The kinetics and voltage dependence of currents were analyzed with non-linear least squares curve fitting using Origin (OriginLab, Northampton, MA, USA).

## Results

### The cloned human ERG channel current is reduced by the Src-family kinase inhibitor, PP1

First, we used patch-clamp analysis of cloned hERG/Kv11.1 channels to assess their regulation by Src-family tyrosine kinases, and specifically, by Src itself. The biophysical parameters examined were: current amplitude at a wide range of voltages, the voltage dependence of activation and inactivation, and the time constants of opening and closing. For the rapidly inactivating hERG channels, the voltage dependence and kinetics of inactivation were also measured. To analyze hERG regulation, the voltage protocols were determined by the unique gating properties of hERG channels (for reviews, see references [31], [32]). At very negative membrane potentials hERG channels remain closed and inactivation is removed. In response to a sufficiently depolarizing step, hERG channels open very slowly but inactivate with fast kinetics (c→o→i). Then, in response to a strong hyperpolarizing step, channels quickly recover from inactivation and briefly transition through the open state (i→o), which yields a characteristic tail current, and then slowly closes (o→c).

**Figure 1:**
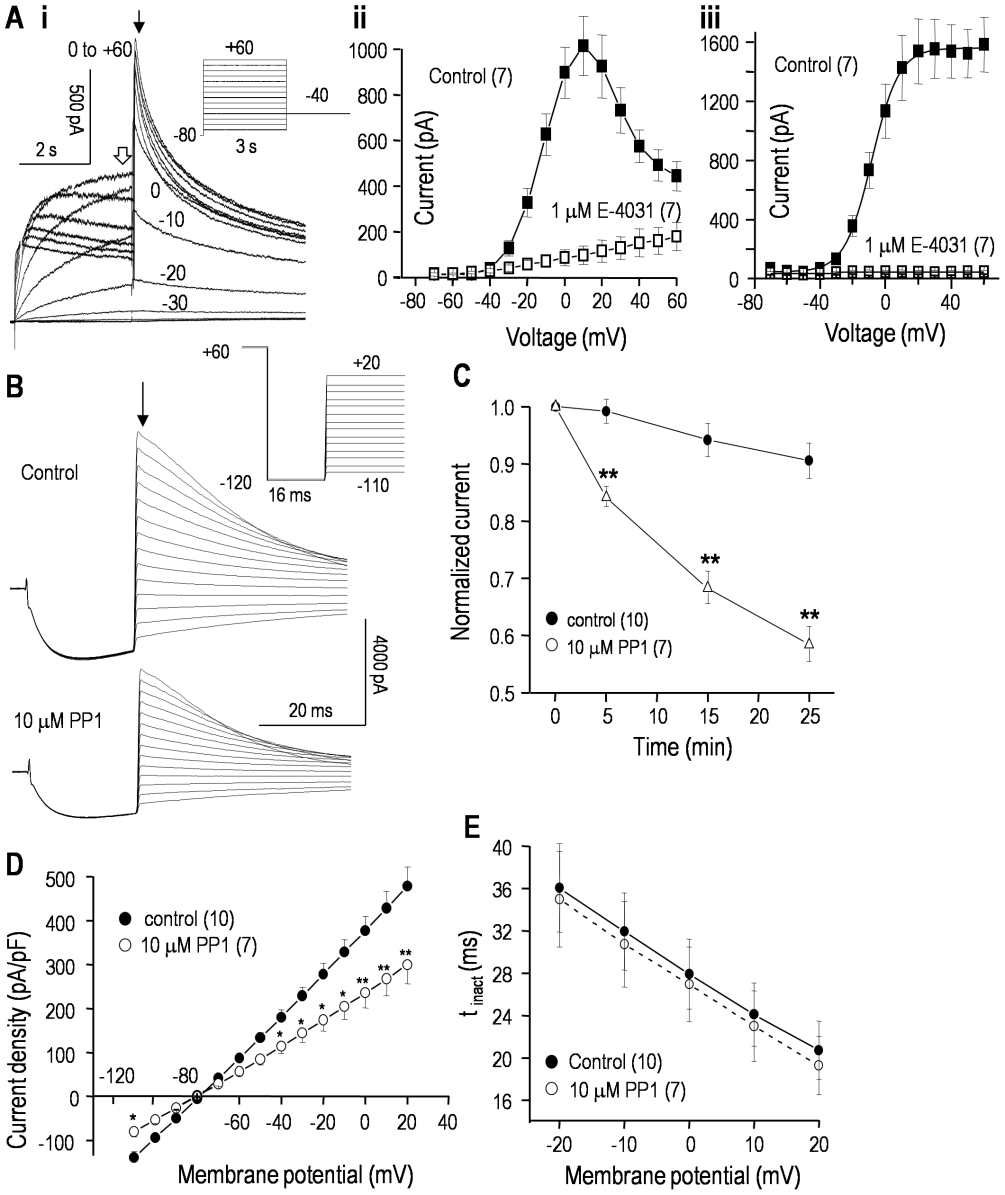
The hERG current in stably transfected HEK293 cells is reduced by the Src-family tyrosine kinase inhibitor, PP1. **A. i.** Representative current traces in response to the voltage-clamp protocol shown in the inset. From the holding potential of −80 mV, 3-s long depolarizing steps were applied between −70 and +60 mV (10-mV increments). This opens the channels, but at positive potentials, some inactivation is seen (c→o→i). The voltage was then stepped to −40 mV to remove inactivation (i→o) and monitor outward tail currents. **ii.** The current amplitude at the end of each 3-sec long depolarizing step (open arrow in Ai) was used to construct a current-versus-voltage (I–V curve) before and after perfusing the hERG blocker, 1 µM E-4031, into the bath. **iii.** Summary of instantaneous I–V relations from tail currents (closed arrow in Ai) before and after adding E-4031. In all graphs, the values are mean±SEM for the number of cells in parentheses. **B.** Representative whole-cell currents are shown before and 25 min after adding 10 µM PP1 to the bath. From a holding potential of −80 mV, a 1 s long pulse to +60 mV was used to activate and then inactivate the channels (c→o→i). Then, inactivation was rapidly removed by a 16 ms long pulse to −120 mV (i→o). Instantaneous tail currents (arrow) during test pulses between −110 and +20 mV (10-mV increments) were used to measure the open-channel current-*vs*-voltage (I–V) relationship (summarized in D) and inactivation kinetics (summarized in E). **C.** PP1 reduces the hERG current. To monitor time-dependent changes, the instantaneous tail current at +20 mV was repeatedly measured in control cells and in separate cells exposed to 10 µM PP1. Each current was normalized to its initial value (measured at 3–4 min after starting the recording). Significant differences between control and PP1-treated cells are indicated as *p<0.05; **p<0.01. **D.** Open-channel I–V relations from instantaneous tail currents (as in panel B) were recorded at 25 min in control cells and in separate cells exposed to 10 µM PP1. For each cell, the current was normalized to cell capacitance to yield current density (pA/pF). **E.** The time constant of inactivation (τ) was measured after 25 min in control cells or with 10 µM PP1 in the bath. That is, for test pulses between +20 and −20 mV, the decay of the tail current (o→i; as in panel B) was fitted to a mono-exponential function: *I_t_* = AMP*exp(−*t/τ*), where *I_t_* is the outward current at time *t* and *AMP* is the initial current amplitude. Each fit was begun at the time indicated by the arrow in panel B.

Figure 1A shows the basic features of hERG currents in the stably transfected HEK293 cells. Depolarizing steps evoked outward currents that initially increased with depolarization, but then some inactivation is evident with further depolarization. The summarized current-versus-voltage (I–V) curves of the current at the end of each step (Fig. 1Aii) show the typical bell shape and inhibition by the hERG blocker, E-4031. The decrease at positive potentials reflects inactivation [31], [32]. The large outward tail currents upon return to −40 mV represent channels that were open at the end of each test voltage step between −70 and +60 mV, and were used to construct instantaneous I–V curves (Fig. 1Aiii) and conductance-versus-voltage relations (see below). The large outward tail currents (∼1600 pA) were fully blocked by E-4031.

hERG regulation by tyrosine kinase action was first assessed by monitoring the current in each cell before and after bath addition of the membrane-permeant Src-family kinase inhibitor, PP1 [33]. Currents were compared with separate control cells exposed to 0.1% DMSO, the solvent for PP1. Figure 1B shows the protocol used to monitor the effects of PP1 on the current amplitude, reversal potential, open-channel current-vs-voltage (I–V) relationship and maximal conductance. Instantaneous tail currents were used to monitor open-channel properties. Tail currents were evoked using a triple-pulse protocol, where a strong depolarization from −80 to +60 mV allowed the channels to slowly open, and quickly inactivate. Then, inactivation was rapidly relieved during a very brief pulse to −120 mV, and tail currents were monitored during test pulses between −110 and +20 mV. During each test pulse, the tail current decayed due to inactivation at depolarized potentials and closing at hyperpolarized potentials. As seen in Figure 1C, there was very little rundown of hERG current during the 25 min test period (<5% decrease) in control cells, compared with a time-dependent reduction by PP1 (∼42%). Steady-state inhibition by PP1 was not determined because of the need for very long recordings, which became unstable. Figure 1D shows the hERG current density over a wide range of voltages. Open hERG channels do not rectify [31], [32] and the I–V curve was linear, as expected. After correcting for the ∼5 mV junction potential, the current reversed at the theoretical Nernst potential for K^+^ (−85 mV) and this was unaffected by PP1. [NB: PP1 did not activate endogenous currents in non-transfected HEK293 cells (not shown)]. Inactivation rates were determined from tail current decay times between −20 and +20 mV, and were unaffected by PP1 (Fig. 1E). Thus, it appears that the reduced current after PP1 is not due to faster inactivation in this voltage range.

### Inhibition of Src-family kinases reduces hERG window current without apparent effects on voltage dependence or kinetics

**Figure 2:**
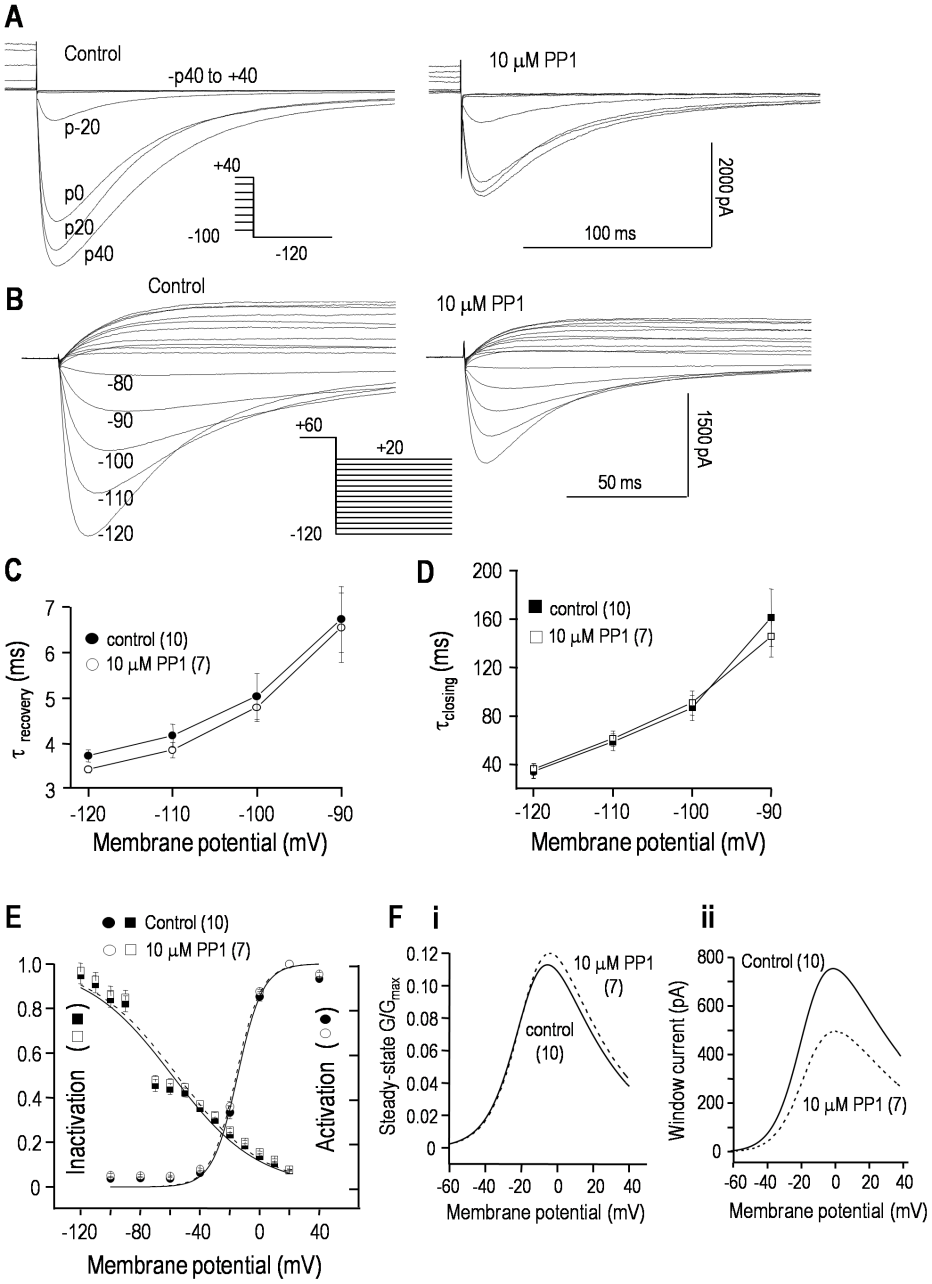
PP1 reduces the hERG window current without apparently affecting the voltage dependence or kinetics. Whole-cell hERG currents in stably transfected HEK293 cells, with representative current traces in response to the voltage-clamp protocols shown as insets. For all panels, control recordings at 25 min are compared with 25 min after adding 10 µM PP1 to the bath. **A.** The voltage dependence of activation was monitored by varying the 6 s long pre-pulse between −100 and +40 mV (20 mV increments) (c→o→i). Then, the maximal inward current was measured during a test pulse to −120 mV, which rapidly relieved inactivation (i→o) and revealed transient hERG currents before the channels slowly closed (o→c). The peak amplitudes were used to construct activation-*vs*-voltage curves (summarized in panel E). **B.** A single protocol was used to monitor the voltage dependence of inactivation, and the time courses of recovery from inactivation and channel closing. From a holding potential of −80 mV, a 1 s-long pre-pulse to +60 mV activated and then inactivated the channels (c→o→i). Then, test pulses were applied between −120 and +20 mV (10 mV increments). The rapid increase in inward currents reflects recovery from inactivation (i→o; summarized in C), and the subsequent slow decline reflects closing (o→c; summarized in D). The maximal current at each test voltage was used to construct inactivation-*vs*-voltage curves (summarized in panel E). **C, D.** Time constants (τ) of recovery from inactivation (C) and closing (D) are summarized. For test potentials between −120 and −90 mV, the currents (as in panel B) were fitted to a dual exponential (rising and falling) function: *I_t_* = *I_o_*+(AMP_r_*exp(−*t/τ_r_*)+AMP_d_*exp(−*t/τ_d_*)). The values are presented as mean±SEM for the number of cells in parentheses. **E.** Activation-*vs*-voltage curves were obtained by fitting the peak currents (examples in panel A) to a Boltzmann equation: *G/G_max_* = 1/(1+exp[(*V_m_*−*V_1/2_*)/*k*]), where *V_m_* is membrane potential, *V_1/2_* is the potential at which *G/G_max_* = 0.5, and *k* is the slope factor. The degree of inactivation as a function of voltage (data as in panel B) was calculated from: *I*/(*G*_slope_(*V_m_*−*E*_K_)), where I is the peak current at each voltage, *V_m_* is the test potential, *E*_K_ is the K^+^ Nernst potential, and *G*_slope_ is the maximal slope conductance, calculated from a linear fit to the I–V relation between −120 and −90 mV (not shown). **F.** The window current was calculated by multiplying the results of each fitted equation (panel E) by the maximal whole-cell conductance and dividing by the driving force (V_m_−E_K_).

In principle, Src-family kinase inhibition by PP1 could affect the channel kinetics, and decrease the current by decreasing overall channel activity, or evoking a positive shift in the voltage dependence of activation or a negative shift in inactivation. Data in Figure 2 show that an overall decrease in channel activity is the most likely cause (see figure legend for details of voltage protocols). Representative recordings of hERG activation show that a 25 min exposure to 10 µM PP1 decreased the current by ∼45% (Fig. 2A) but did not obviously affect the kinetics, which are quantified below. Figure 2B shows representative current traces and the voltage protocol used to assess channel inactivation and the time courses of channel closing and recovery from inactivation. The maximal conductance, calculated from a linear fit to the currents between −120 and −90 mV, decreased from 86.6±7.9 nS (n = 10) in control cells to 54.8±4.2 nS (n = 7) 25 min after exposure to PP1 (*p*<0.01). This reduction was independent of voltage: about ∼60% at all voltages between −110 and +20 mV. Neither the time constant of recovery from inactivation (Fig. 2C) nor channel closing (Fig. 2D) were affected by PP1 treatment. In addition, the activation-*vs*-voltage and inactivation-*vs*-voltage relations, calculated as shown in Figure 2E, did not differ between control and PP1-treated cells. The half-maximal voltage (*V*_1/2_) for activation was −13.4±0.9 mV (control) *vs* −12.9±0.8 mV (PP1; *p*>0.5), and the slope factor (*k*), describing the steepness of the voltage dependence, was 7.8±0.4 mV (control) *vs* 8.2±0.5 mV (PP1; *p*>0.5). For inactivation, *V*_1/2_ was −54.8±3.1 mV (control) *vs* −57.1±3.9 mV (PP1; *p*>0.2), and the slope factor was 28.8±2.6 mV (control) *vs* 27.7±2.3 mV (PP1; *p*>0.2). Taken together, these data show that PP1 reduces the current without apparently affecting the kinetics or proportion of channels active at each membrane potential, but an effect on the single-channel conductance cannot be ruled out.

Because the contribution of hERG channels to non-excitable cells (e.g., cancer cells) is largely determined by the amount of current flowing at their resting potentials, we also examined the ‘window current’. As seen in Figure 2E, the inactivation and activation curves overlap and delineate a voltage range (about −40 to +20 mV) where channels are opened but not inactivated. The two Boltzmann equations were multiplied to assess the voltage window in which channels are expected to be tonically active (Fig. 2Fi); the largest conductance was at about 0 mV. However, the size of the window current depends on the maximal conductance, which was ∼42% lower after PP1 treatment and hence, the window current amplitude was smaller over a wide voltage range (Fig. 2Fii).

### hERG is regulated specifically by Src tyrosine kinase

**Figure 3:**
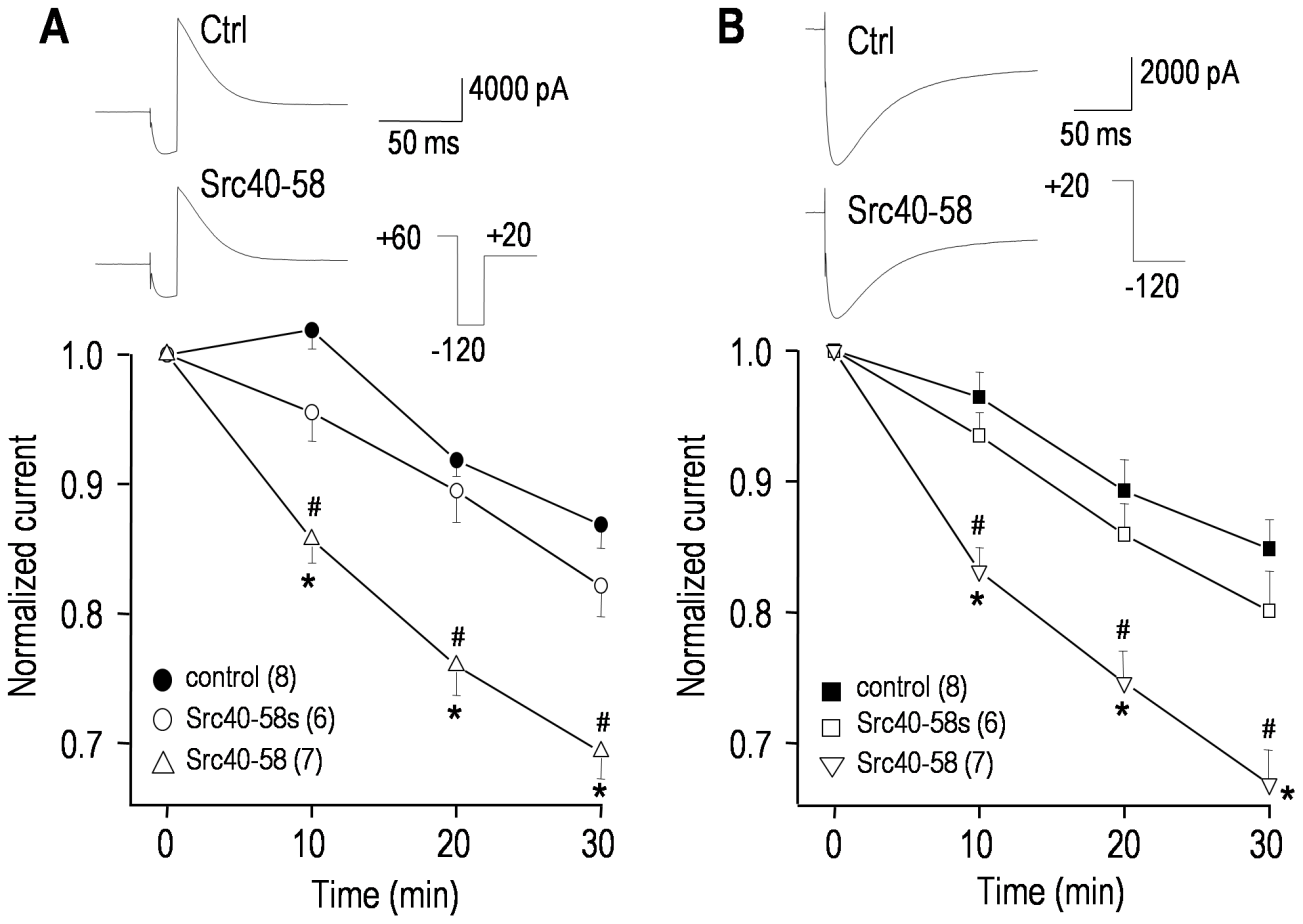
Src tyrosine kinase regulates the hERG current. Currents were compared between cells recorded with pipette solutions containing saline (to control for spontaneous changes), 100 µg/mL of a Src-inhibitory peptide (Src40-58), or 100 µg/mL of a scrambled version of the same peptide (Src40-58s). For each cell, the voltage protocol was repeated at 0, 10, 20 and 30 min of recording; and the representative current traces (upper panels) are from 2 control cells and 2 treated cells, all at 30 min. **A.** The voltage protocol was similar to Fig. 1B; i.e., from a holding potential of −80 mV, a 1 s long pulse to +60 mV was applied to activate the channels. Then inactivation was removed by a 16 ms long pulse to −120 mV, and the maximal outward tail current was measured during a test pulse to +20 mV. The lower panel summarizes the time course, measured from the maximal tail current, normalized to its initial value at 3–4 min after establishing a recording. **B.** Following a 6 s long pre-pulse to +20 mV to activate the channels, the maximal inward tail current was measured during the test pulse to −120 mV (similar protocol to Fig. 2A). The summarized time course in the lower panel was constructed as in panel A. Significant differences for Src40-58 are shown compared with controls (*p<0.05) or the scrambled peptide (^#^ *p*<0.05).

The reduction in hERG conductance by PP1 implicates a Src-family kinase; thus, we next addressed whether Src itself regulates the current. To selectively interfere with Src-target protein interactions, a peptide consisting of Src amino acids 40-58 (Src40-58) was added to the pipette solution, and compared with controls containing a scrambled version of the same peptide (Src40-58s) [16], [34] or saline alone. As each peptide diffused into the cell and exerted its actions, we monitored time-dependent changes in the current (Fig. 3). The Src40-58 peptide reduced the hERG current to a similar extent as PP1 (compare with Fig. 1C), regardless of the voltage protocol used; whereas, the scrambled peptide did not differ from the saline controls. By 30 min, the maximal conductance decreased from 87.2±7.1 nS (saline) to 69.6±5.5 nS (Src40-58; *p*<0.05). From the representative current traces, there were no apparent changes in the kinetics of closing, inactivation or recovery from inactivation, nor did the voltage dependence of activation or inactivation change (not shown). Thus, the effects of Src40-58 were essentially identical to PP1, and as discussed later, this suggests that inhibiting tyrosine phosphorylation by Src reduces the number of active hERG channels and/or their open probability.

### The proto-typical hEAG1 channel is also regulated by a Src-family kinase

**Figure 4:**
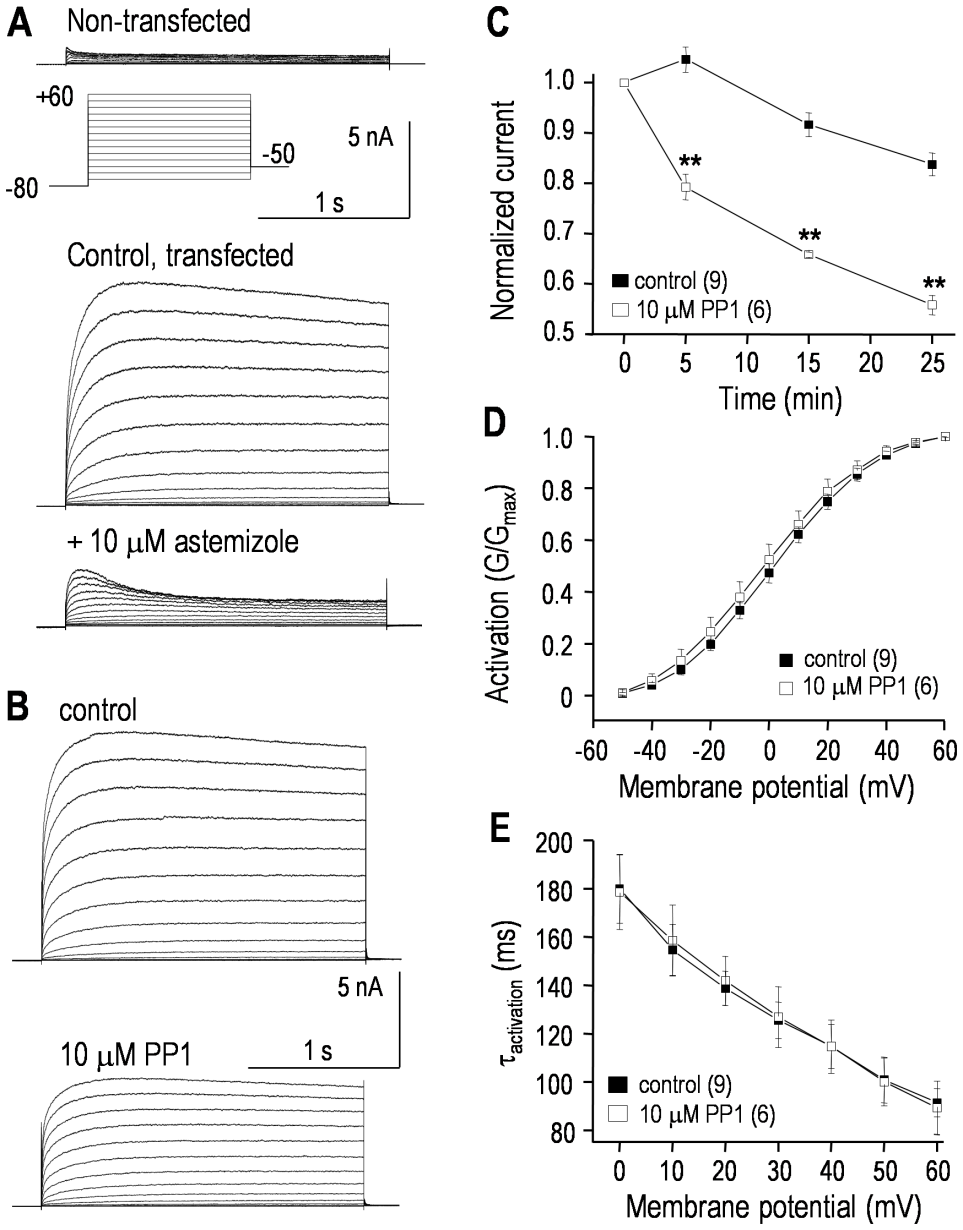
The hEAG1/Kv10.1/KCNH1 current is regulated by a Src-family kinase. Whole-cell recordings of hEAG1 currents in transiently transfected HEK293 cells. The same voltage-clamp protocol (inset) was used for all recordings; i.e., from a holding potential of −80 mV, 1 s-long voltage steps were applied between −70 and +60 mV (in 10-mV increments). **A.** Typical recordings from a non-transfected HEK293 cell, and a hEAG1-transfected cell before and 5 min after adding the EAG blocker, astemizole. Note that the currents are plotted on the same scale. **B.** Representative hEAG1 currents from a transfected cell recorded 3–4 min after establishing a whole cell recording; and 25 min after bath addition of the Src-family kinase inhibitor, PP1. **C.** Summary of time-dependent inhibition of hEAG1 currents by PP1. The voltage protocol was applied every 5 min, and the peak current measured at +60 mV was normalized to its initial value. Significant differences are indicated: **p<0.01. **D.** To obtain activation -*vs*-voltage curves, the peak current during each voltage step was divided by the driving force (*V_m_*–E_K_) to calculate conductance, and then normalized to the largest conductance (i.e., at +60 mV). Each conductance-*vs*-voltage curve at 25 min was fitted to a Boltzmann equation: *G/G_max_* = 1/(1+exp[(*V_m_*−*V_1/2_*)/*k*]), where *V_m_* is membrane potential, *V_1/2_* is the potential at which *G/G_max_* = 0.5, and *k* is the slope factor. The summarized curves are for control cells and separate PP1-treated cells. **E.** Time constants (τ) for hEAG1 current activation were measured for each test pulse by fitting a mono-exponential function: *I_t_* = AMP*exp(−*t/τ*), as in Fig. 1E.

Representative current traces show the very small endogenous outward current in non-transfected HEK293 cells [35] and typical large hEAG1 currents in transiently transfected cells (Fig. 4A). The outward current was 168±20 pA in non-transfected cells while, in hEAG1-transfected cells, the peak current at +60 mV was 10.7±1.1 nA. As expected, the current in EAG-transfected cells was reduced by the hEAG1 blocker, astemizole [36]. With 10 µM astemizole, there was ∼90% block of the current measured at the end of each pulse; i.e., a reduction from 9.8±1.1 to 1.2±0.1 nA at +60 mV (compared with the 168 pA current in non-transfected cells). Because hEAG1 does not rapidly inactivate, a simpler voltage-clamp protocol was used to examine regulation of hEAG1 current by the Src-family inhibitor, PP1. A very negative holding potential (−80 mV) was used to remove inactivation, and then depolarizing steps were applied to activate the current. Representative traces show that there was very little inactivation during the 1-s long steps, and that bath addition of 10 µM PP1 reduced the current without affecting the slow inactivation (Fig. 4B). PP1 gradually reduced the hEAG1 current, which reached ∼44% by 25 min, compared with ∼14% spontaneous rundown in time-matched control cells (Fig. 4C). Again, steady-state inhibition by PP1 was not determined because much longer recordings became unstable. Activation (conductance)-*vs*-voltage curves were constructed from the peak current during each voltage step, and fitted to a Boltzmann equation (see figure legend). PP1 did not alter the voltage dependence of activation (Fig. 4D); *V*_1/2_ was 2.1±0.1 mV (control) *versus* 1.3±0.1 mV (PP1; *p*>0.2) and the slope factor was 15.1±0.9 mV (control) *versus* 15.2±1.1 (PP1; *p*>0.2). As apparent from the similar kinetics of the representative currents, PP1 had no obvious effect on the inactivation rate, and the time constant of activation as a function of voltage was not changed (Fig. 4E). Because of the very negative holding potential (−80 mV), the decrease in current during each step depolarization was not due to inactivation; thus, we did not compare the steady-state inactivation curves.

### The ITIM motif of hERG interacts with, and activates SHP-1 tyrosine phosphatase

**Figure 5:**
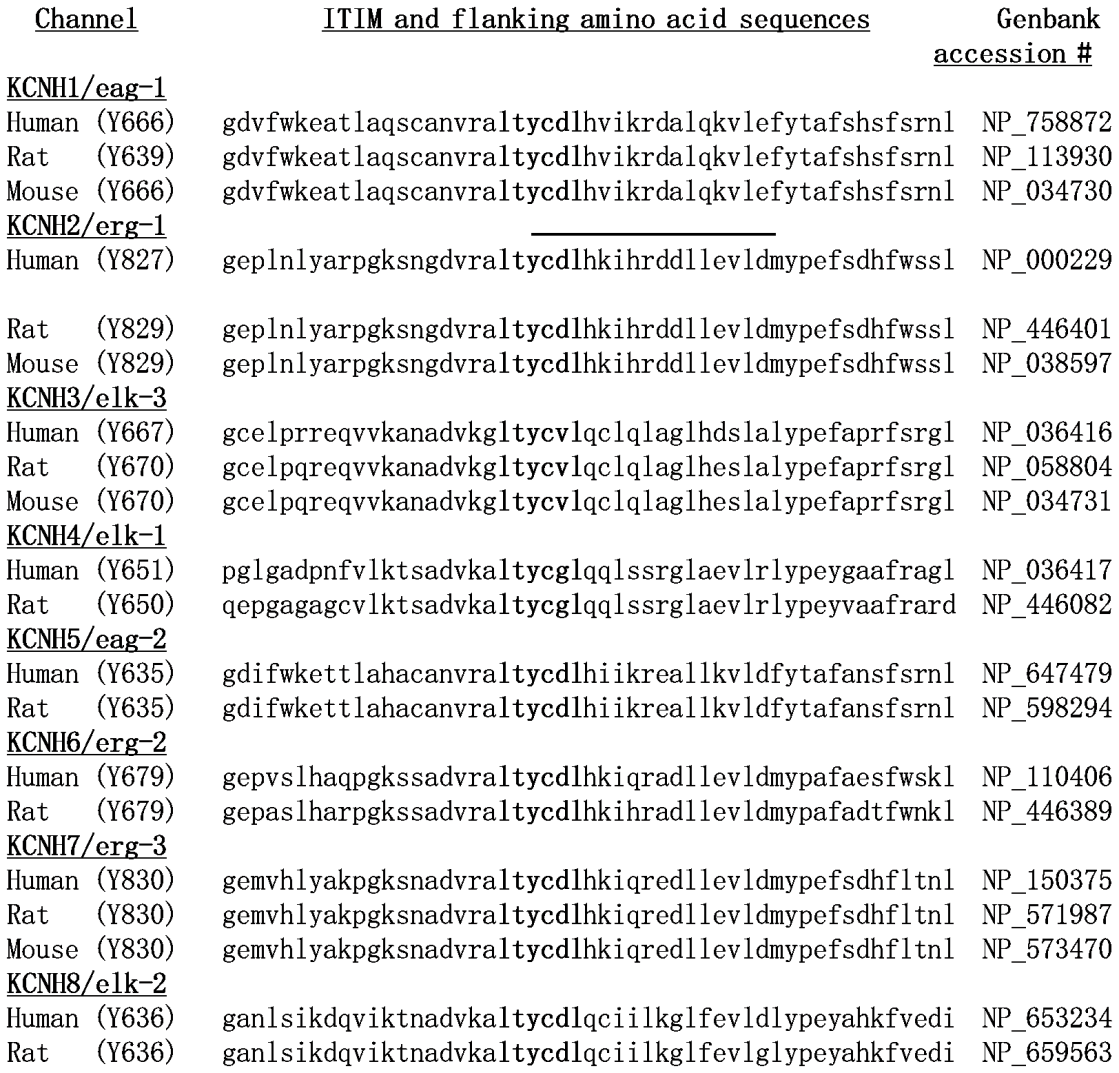
EAG-family members contain a conserved ITIM motif. Sequence alignments of C-termini for the EAG family of K^+^ channels show each immuno-receptor tyrosine inhibitory motif (ITIM) in bold, with its potentially phosphorylated tyrosine residue in parentheses beside the channel name. Also shown for hERG/Kv11.1/KCHN2 is the sequence of the 15 amino acid peptide made for use in pull-down assays or deleted in the ΔITIM mutant channel (line above the sequence).

Based on the regulation of hERG and hEAG1 by Src-family kinases, we were next interested in the possibility that a specific tyrosine phosphatase reciprocally regulates these channels. We previously found that SHP-1 regulates the ERG-like current in the rat MLS-9 microglial cell line [23]. At that time, we noted that the rERG sequence contains an ‘immuno-receptor tyrosine inhibitory motif’ (ITIM) consensus sequence (Iso/Val/Leu-X-Tyr^829^-X-X-Leu/Val). Here, we realized that all known members of the EAG super-family of K^+^ channels [37] contain an ITIM consensus sequence (Iso/Val/Leu-X-Tyr-X-X-Leu/Val) in their cytoplasmic C-terminus (Fig. 5).

**Figure 6:**
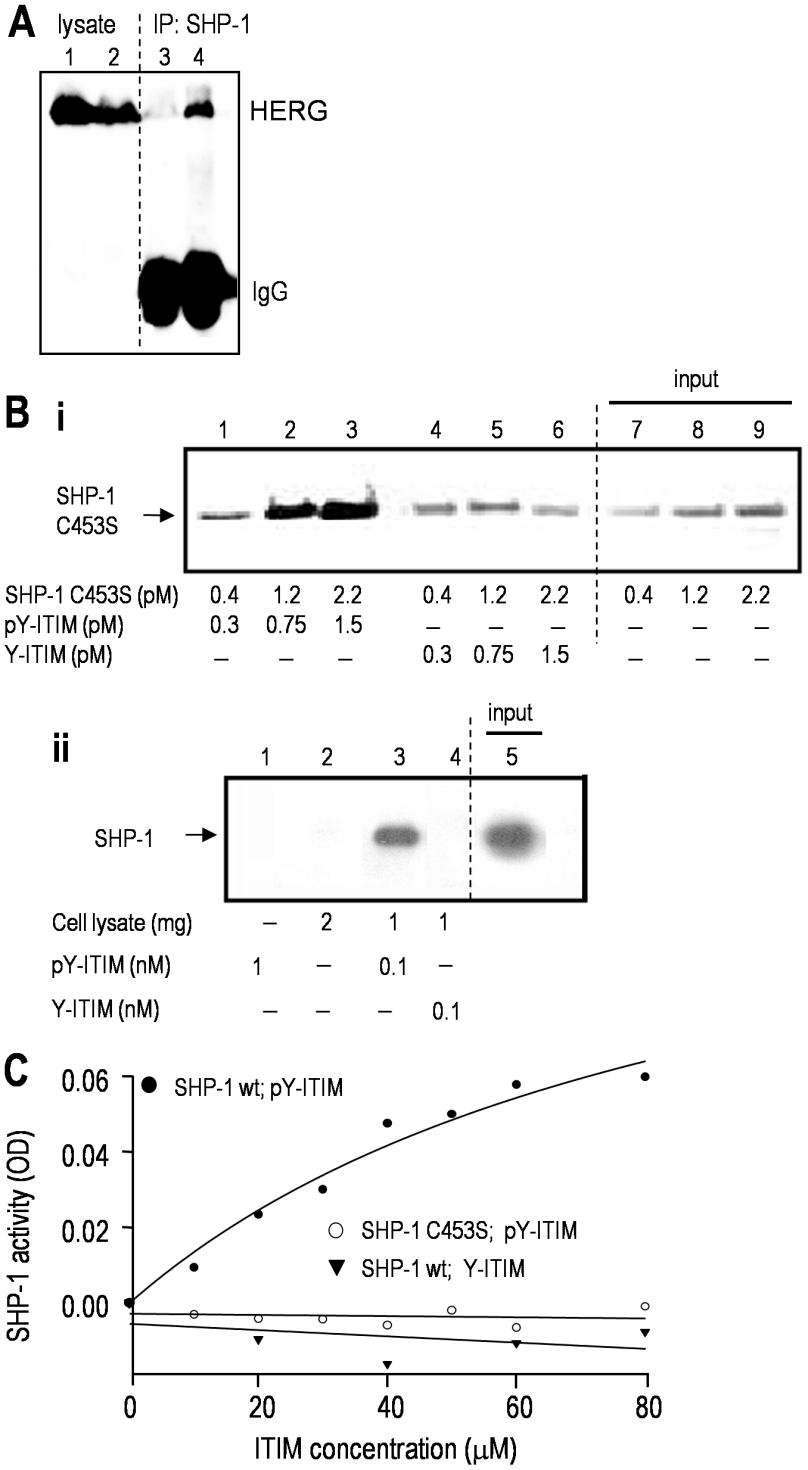
The ITIM of hERG/Kv11.1 binds to, and trans-activates SHP-1 tyrosine phosphatase. **A.** The ITIM motif of hERG is required for its interaction with SHP-1. HEK293 cells expressed either wt hERG (lanes 1&4) or ΔITIM hERG (lanes 2&3). Immunoprecipitation: anti-SHP-1 antibody; Western blots: anti-hERG antibody. The lysate lanes show that channel protein expression was not affected by ITIM deletion. **B.** Tyrosine phosphorylation of the hERG ITIM is required for its direct binding to SHP-1. **i.** Purified recombinant SHP-1 C453S protein was incubated with increasing concentrations of the phosphorylated (pY-ITIM; lanes 1–3) or non-phosphorylated (Y-ITIM; lanes 4–6) ITIM-hERG peptide (see Methods). The peptides, which were biotinylated, were pulled down with avidin-coated beads. The Western blot was probed with anti-SHP-1 antibody, and lanes 7–10 show the input SHP-1 C453S protein. **ii.** Pull-down of endogenous SHP-1. Lysates from MLS-9 cells, which express SHP-1 (lane 5; 80 µg of input lysate), were incubated with each biotinylated ITIM-hERG peptide, which was pulled down with avidin-coated beads and probed for SHP-1, as above. **C.** The SHP-1 enzyme is activated by the ITIM of hERG. Phosphorylated or non-phosphorylated ITIM-hERG peptides were incubated for 3 h with 0.2 µg of wt SHP-1 in a buffer containing 10 mM *p*NPP (*p*-nitrophenyl phosphate) as the substrate (see Methods). Similarly, the tyrosine phosphorylated ITIM-hERG peptide was incubated with SHP-1 C453S.

Next, we determined that this motif is necessary for the interaction of hERG with SHP-1 tyrosine phosphatase. That is, when HEK293 cells were transfected with myc-tagged hERG, or with hERG in which the ITIM motif and several flanking amino acids were deleted (ΔITIM-hERG), only the full-length hERG protein co-immunoprecipitated with SHP-1 protein (Fig. 6A). The loading controls, input lanes 1 and 2, and the IgG bands in lanes 3 and 4 show that ΔITIM-hERG was well expressed. A crucial first line of evidence that the ITIM is functional is that it must be tyrosine phosphorylated in order to bind to SHP-1, and pull-down assays (Fig. 6B) show this to be the case. SHP-1 interaction with its substrates can be transient [38]. Thus, for the binding assay, we exploited a ‘substrate-trapping’ SHP-1 mutant (SHP-1 C453S) that has a higher than normal binding affinity but lacks enzymatic activity SHP-1, so the binding to target proteins can withstand immunoprecipitation [39]. The tyrosine-phosphorylated ITIM-hERG peptide pulled down SHP-1 C453S in a concentration-dependent manner (Fig. 6Bi; lanes 1–3); whereas, little or no interaction was seen with the non-phosphorylated hERG peptide (lanes 4–6). Next, to assess whether the same interaction occurs with native SHP-1, we used a microglial cell line that expresses the hematopoietic version of SHP-1 (MLS-9) and found that only the tyrosine-phosphorylated ITIM-hERG peptide pulled down endogenous SHP-1 (Fig. 6Bii). Finally, we assessed the functionality of the hERG ITIM by examining its ability to trans-activate the enzymatic function of SHP-1 and convert the substrate, *p*-nitrophenyl phosphate, to *p*-nitrophenol [29]. Only the tyrosine-phosphorylated ITIM-hERG peptide increased the phosphatase activity of wt SHP-1, and it did so in a concentration-dependent manner (Fig. 6C). Moreover, as expected, SHP-1 C453S did not exhibit phosphatase activity, even in the presence of the tyrosine-phosphorylated ITIM-hERG peptide. This is apparently the first demonstration that a motif in an ion channel serves as a binding site for, and activates, a tyrosine phosphatase.

### SHP-1 tyrosine phosphatase regulates hERG

**Figure 7:**
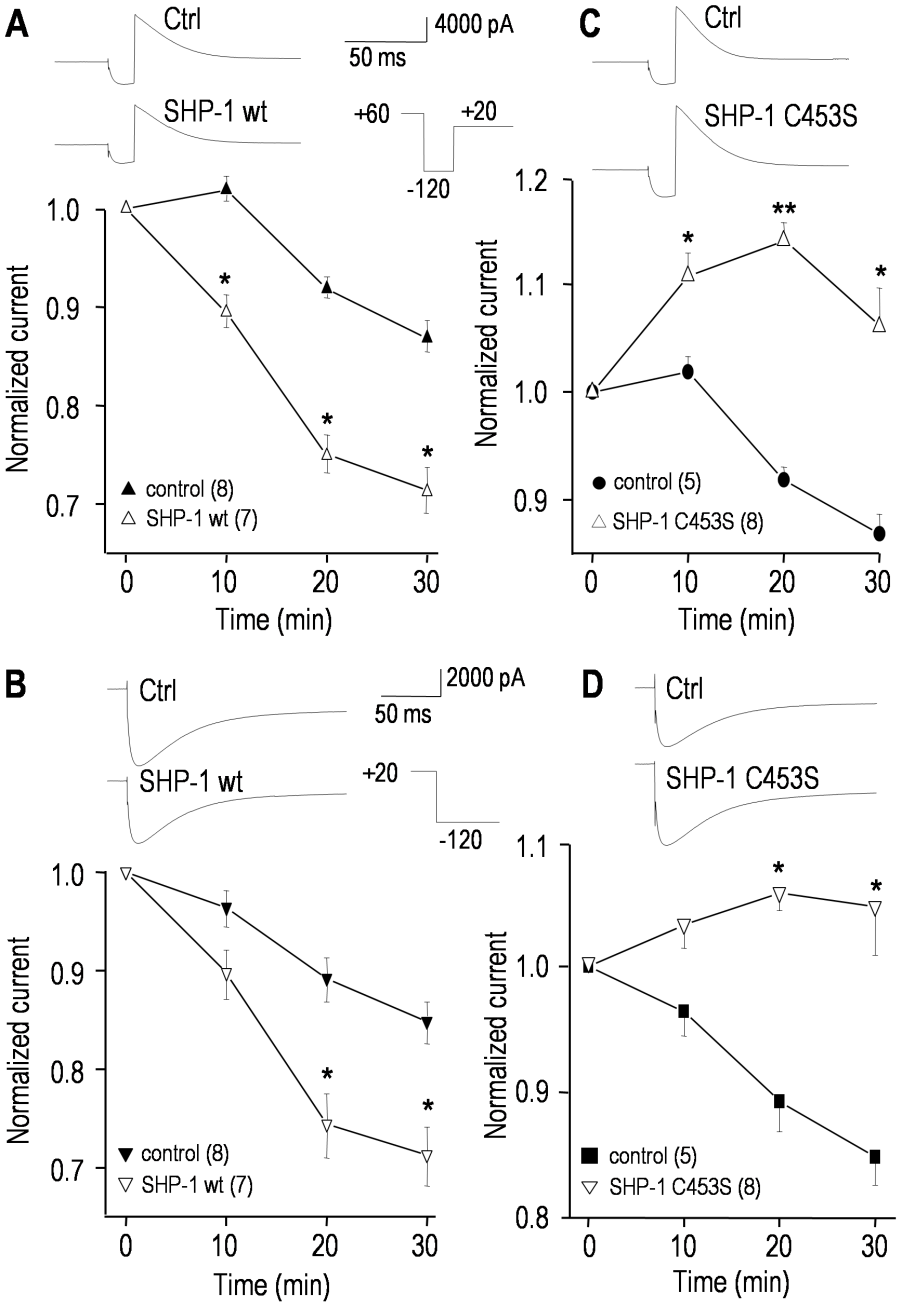
SHP-1 tyrosine phosphatase regulates the hERG current. Currents were compared between cells recorded with pipette solutions containing saline (control), or 100 µg/mL of active recombinant wild-type SHP-1 (**A, B**), or a 3∶1 mixture of the inactive substrate-trapping SHP-1 mutant (SHP-1 C453S; 150 µg/mL) and wild-type SHP-1 (50 µg/mL) (**C, D**). The same voltage protocols and normalization procedures were used as in Figure 3. For each panel, representative current traces from two different cells are shown, and the lower graphs summarize the time-dependent changes in peak currents. Significant differences between control cells and cells containing SHP-1 proteins are indicated: *p<0.05; **p<0.01.

Inhibiting Src action with PP1 or Src40-58 inhibited hERG (Figs. 1–3), supporting the hypothesis that de-phosphorylation of one or more tyrosine residues reduce channel activity. Based on the presence of an ITIM that both binds to, and activates SHP-1 (Figs. 5, 6), we next assessed whether recombinant SHP-1 protein modulates the current. Adding wild-type SHP-1 (an active phosphatase) to the pipette slowly reduced the hERG current (Fig. 7A, B); i.e., the conductance after 30 min was 75.8±5.2 nS (wt SHP-1), compared with 88.1±7.8 nS (control; *p*<0.05). Next, we added an excess of a recombinant substrate-trapping SHP-1 mutant (SHP-1 C453S), which binds to target molecules but cannot de-phosphorylate their tyrosine residues [40], [41], and thus acts as a competitive inhibitor, protecting target proteins from de-phosphorylation. To further ensure that competitive SHP-1 inhibition occurred, SHP-1 C453S was added in the presence of wild-type SHP-1. Not only did SHP-1 C453S prevent wt SHP-1 from inhibiting hERG (Fig. 7C, D), it increased the current compared with the normal spontaneous rundown (Fig. 7A, B). As is apparent in the representative currents, there were no changes in the kinetics or voltage-dependence of activation or inactivation (not shown). The effects of both wild-type and substrate-trapping mutant SHP-1 are entirely consistent with the effects of the Src-family kinase inhibitor, PP1, and the inhibitory Src peptide (Src40-58). Unfortunately, regulation of ΔITIM hERG could not be studied, because there was no current in HEK293 or CHO cells transfected with this construct, despite a robust band in a Western blot from a membrane preparation.

### The hEAG1 current is modulated by SHP-1 tyrosine phosphatase

**Figure 8:**
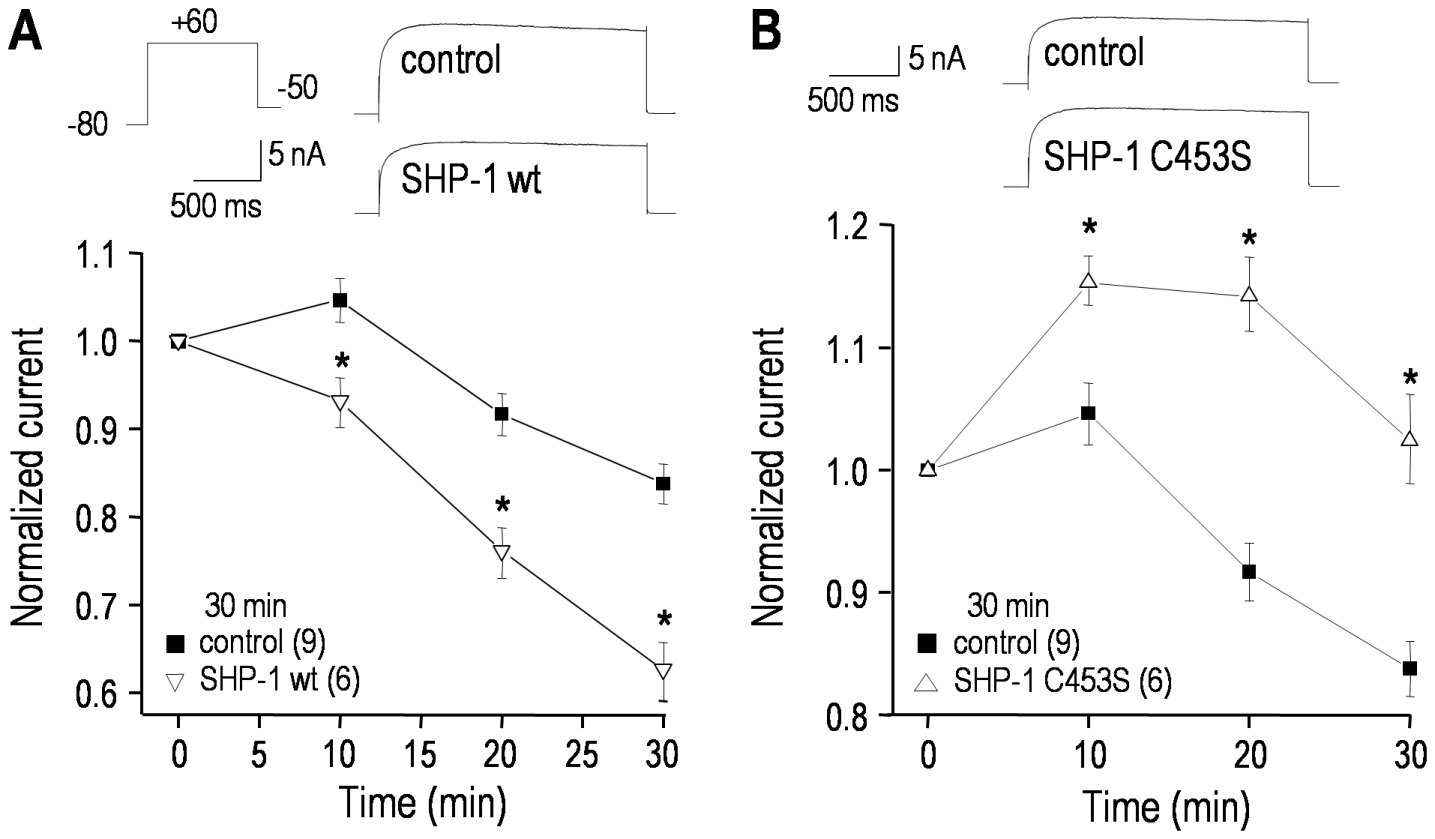
The hEAG1/KCNH1 current is regulated by SHP-1 tyrosine phosphatase. The voltage protocols and normalization procedures were the same as in Fig. 4 (only traces at +60 mV are shown). **A.** Time-dependent effects of adding 100 µg/mL recombinant wild-type SHP-1 protein to the pipette solution. Inset: representative currents from two cells, recorded 30 min after establishing the whole-cell configuration. For each cell, the peak current at +60 mV was repeatedly measured and normalized to its initial value (3–4 min after establishing a recording) for the number of cells indicated (*p<0.05). **B.** Comparison of cells recorded with control pipette solution with those containing a 3∶1 mixture of the inactive substrate-trapping SHP-1 mutant (SHP-1 C453S; 150 µg/mL) and active wild-type SHP-1 (50 µg/mL). Inset: representative currents recorded 30 min after establishing the whole-cell configuration. The normalized peak currents at +60 mV are shown; *p<0.05.

Adding recombinant wild-type SHP-1 protein to the intracellular pipette solution significantly reduced the hEAG1 current (Fig. 8A, B), just as it did for hERG (compare Fig. 7). In contrast, adding an excess of the substrate trapping SHP-1 C453S mutant, which protects target proteins from SHP-1 dependent de-phosphorylation (i.e., favoring tyrosine phosphorylation) significantly increased the hEAG1 current, even in the presence of wt SHP-1 (Fig. 8C,D). As shown above for the hERG current, representative hEAG1 currents show no apparent changes in kinetics or voltage dependence of activation, and this was corroborated by quantitative analyses of *V*_1/2_, the slope factor and the time constant of activation (not shown).

## Discussion

### Summary of findings

There are three salient findings, which will be discussed in detail below. 1. This is the first demonstration that hERG and hEAG1 are regulated by the protein tyrosine phosphatase, SHP-1. Both channels are also regulated by Src, the prototypical member of a large family of non-receptor tyrosine kinases. We found that both currents were decreased by treatments that can reduce tyrosine phosphorylation; i.e., by a tyrosine kinase inhibitor (PP1), by directly inhibiting Src actions, or by applying active recombinant SHP-1 phosphatase. Conversely, inhibiting SHP-1 by applying its inhibitory substrate-trapping mutant increased both currents and abrogated their spontaneous rundown during whole-cell recording. With each treatment, the current amplitude was affected, without apparent changes in the voltage dependence or kinetics.

2. Studies of K^+^ channel regulation often focus on the current amplitude and conductance at very positive potentials, without considering changes in voltage dependence or steady-state ‘window’ current. Consequently, there is a dearth of information about regulation of EAG-family channels that is relevant to their function in non-excitable cells (e.g., immune cells, cancer cells, vascular smooth muscle) or during the repolarization phase of cardiac action potentials (the Q-T interval). We show that the largest tonically active hERG current is at ∼0 mV, and the tyrosine kinase inhibitor, PP1, decreased this steady-state window current. A similar decrease in hEAG1 window current is expected based on the similarity in the biophysics of its inhibition.

3. We have identified a highly conserved and functional protein-protein interaction domain, the ‘immuno-receptor tyrosine inhibitory motif’ (ITIM), within the C-terminus of all known members of the EAG-ERG-ELK family of K^+^ channels. Our results show that the tyrosine-phosphorylated ITIM mediates channel binding to SHP-1 tyrosine phosphatase, and is sufficient to transactivate SHP-1. This is apparently the first demonstration that an ion channel serves as a binding site for, and activator of a tyrosine phosphatase.

### Comparison with previous studies

Most studies of regulation of ERG and EAG channels have addressed the serine/threonine protein kinases, PKA, PKC and CaMKII ([42]–[46]; recently reviewed [47]). However, all known members of the ‘*ether-a-go-go*’ (EAG/ERG/ELK) channel superfamily contain potential sites for tyrosine phosphorylation. Surprisingly little is known about this aspect of regulation. In 2002, in the first and only papers on regulation of native ERG-like currents by tyrosine phosphorylation, we discovered that both Src and SHP-1 regulate the current in the rat microglial cell line, MLS-9 [16], [23]. hERG and hEAG1 channels can exist as hetero-tetramers [48]–[50], and our earlier studies were limited by not knowing the molecular composition of the underlying channels; i.e., MLS-9 cells also express transcripts for the shorter rERG-1B isoform and for rERG-2, which might be differentially regulated. At that time, we showed that rERG1 protein in MLS-9 cells co-immunoprecipitates with Src. While our earlier results were significant, they do not address whether regulation is isoform specific, cell-type specific, or can be extended to the human channels, hERG or hEAG1. Since then, apparently only two papers have addressed regulation of the human isoforms by tyrosine kinases. There are some similarities between these studies but there are also differences in experimental design, and in outcomes, which will be addressed in some detail.

In 2008, it was reported that the tyrosine-kinase linked receptor for epidermal growth factor (EGFR) regulates heterologously expressed hERG channels. The hERG current was decreased by an EGFR inhibitor (AG556) and a broad-spectrum inhibitor of Src-family tyrosine kinases (PP2) [24]. In 2012, a second paper from the same group showed that only the EGFR inhibitor decreased the hEAG1 current [25]. Using the Src-family inhibitor, 10 µM PP1 (a pyrazolopyrimidine related to PP2), we observed a similar inhibition of heterologously expressed hERG and hEAG1 channels (present study), and the native ERG-like current in MLS-9 cells [16], and in both studies, the decrease occurred over several minutes. For MLS-9 cells, there was a similar time course of reduction when ATP was omitted from the intracellular solution, suggesting that de-phosphorylation was occurring during the recording. In the present study, the inhibition by PP1 had not reached steady state by 25 min but there was very little spontaneous rundown over this period. The two recent papers using 10 µM PP2 reported inhibition of hERG [24] but no effect on hEAG1 [25]. The efficacy for inhibiting Src is similar for PP1 and PP2, with IC_50_ values of 0.054 and 0.036 µM, respectively [33]. At 10 µM, PP1 and PP2 can inhibit other members of the Src-family (e.g., Lck) and some other PTKs (e.g., RIP2, CK1δ), and PP1 (but not PP2) also inhibits C-terminal Src kinase (CSK) [33]. We present direct evidence that Src regulates hERG; i.e., the Src-specific inhibitory peptide (Src40-58) similarly reduced both cloned hERG1 (present study; hEAG1 not tested) and the ERG-like current in MLS-9 cells [16]. The previous and present data do not rule out roles for other tyrosine kinases and, in future, it would be valuable to further address this, and the apparent cell-dependent regulation of these channels.

Here, we show that both hERG and hEAG1 currents are decreased by applying an active recombinant SHP-1 phosphatase to reduce tyrosine phosphorylation. Conversely, there is an important implication of our finding that both currents increased when inhibitory mutant SHP-1 (SHP-1 C453S) protein was present in the pipette (at the peak of their responses both were ∼25% larger than control cells). SHP-1 C453S works by shielding target tyrosine phosphorylation sites from de-phosphorylation. The time-dependent increase in current suggests that tyrosine phosphorylation occurs during the recording, and that these channels were not maximally phosphorylated in HEK cells. While we specifically examined the effects of SHP-1 tyrosine phosphatase, a different result was seen in the two previous studies that used the broad-spectrum tyrosine phosphatase inhibitor, orthovanadate. There was no increase in hERG and hEAG1 currents, and it was proposed that basal tyrosine phosphorylation might be saturated [24], [25]. Another difference is that our control currents were up to three times larger, which might affect the ratio of channel protein to potential endogenous modulators.

We had previously shown that endogenous SHP-1 can bind to the native rat ERG1 isoform and to endogenous Src in MLS-9 cells [23]. Now, we have identified the site where SHP-1 binds to the cloned hERG1 channel; the tyrosine-containing ‘immuno-receptor tyrosine inhibitory motif’ (ITIM; Leu-Thr-Tyr(P)-Cys-Asp-Leu). The ITIM is an SH2-binding sequence that can be tyrosine phosphorylated by Src, and is then thought to recruit and activate SHP tyrosine phosphatases to de-phosphorylate residues in target proteins [51]. Of note, then, only when tyrosine phosphorylated, the ITIM region of hERG1 activated SHP-1. Once activated, it is possible that SHP-1 dephosphorylates the ITIM site and is self-limiting or that it acts on other pTyr sites, including those phosphorylated by Src, Src-related kinases, and tyrosine receptor kinases. Because a highly conserved ITIM is present within the cyclic nucleotide binding domain (cNBD) in the C-terminus of all channels in the EAG family; it is likely to be a SHP-1 binding and activation motif in other EAG channels.

Together, the present results show that tyrosine phosphorylation increases, and de-phosphorylation decreases the amplitude of cloned hERG and hEAG1 currents. PP1, Src40-58, and wild-type SHP-1, which are expected to reduce phosphorylation, decreased the currents. In contrast, the current was increased by the substrate-trapping SHP-1 C453S mutant, which inhibits de-phosphorylation and thus increases phosphorylation. For all treatments, changes in the current amplitude were time-dependent over a course of several minutes; we quantified most effects at 25 min after treatment. This is expected because modulators of protein phosphorylation are expected to take some time, and the time course is consistent with our earlier papers on regulation of the native ERG-like current in MLS-9 cells [16], [23]. In our other K^+^ channel regulation studies, activators and inhibitors of kinases and phosphatases exerted their effects over many minutes [52]–[54].

Other differences between the existing studies suggest cell-specific regulation of the channels. In principle, current amplitudes can be altered by changing the number of active channels or by several biophysical mechanisms; i.e., altered voltage dependence, kinetics, or open probability. Here, hERG regulation by Src and SHP-1 did not apparently affect the voltage dependence or kinetics of activation or inactivation. Instead, by affecting the overall conductance, the window current was changed, and this suggests that Src and SHP-1 affect the number of active channels and/or their open probability. The present results are consistent with, and extend, the 2008 study showing that Src inhibition did not change the voltage-dependent activation of hERG [24]. Changes in unitary conductance seem unlikely to account for the large decrease in current seen with PP1, Src40-58, and wt SHP-1, and the increase with SHP-1 C453S. The currents changed (decreased, increased) in a time-dependent manner, while most changes in single-channel conductance require channel mutations or temperature changes. While we cannot rule out changes in channel trafficking, we also think it unlikely. The changes in current occurred during whole-cell recordings at room temperature, were relatively rapid (minutes), and would have necessitated both channel removal (PP1, Src40-58, wt SHP-1) and insertion (SHP-1 C453S).

In contrast to the studies on cloned hERG and hEAG1, regulation of the native ERG-like current in MLS-9 cells involved changes in both conductance and voltage dependence. For instance, when MLS-9 cells were transfected with active v-Src, the current amplitude increased and the voltage dependence of activation was altered to facilitate opening, which together increased the steady-state window current. Conversely, active SHP-1 phosphatase decreased the window current by reducing the conductance and positively shifting the voltage dependence of activation (but not inactivation). Thus, tyrosine phosphorylation might have differential effects on the voltage dependence of the currents in different cell types. While it is conceivable that different ERG isoforms account for this, an argument against this interpretation is the lack of altered voltage dependence of the more distantly related hEAG1 channels (present study). Several alternative possibilities are worth addressing in future studies. (i) The different cells in which native ERG and EAG channels exist might differ in expression of accessory molecules that affect channel function; e.g., KCNE1–3 [55]–[58]. (ii) Cells undoubtedly differ in amounts and/or activity of Src-family kinases and tyrosine phosphatases; thus, the baseline level of tyrosine phosphorylation likely differs. In this regard, it is interesting that MLS-9 cells contain abundant SHP-1 (present study; [23]), which is absent from the HEK293 cells used for hERG transfection (present study). (iii) Crosstalk in regulation by kinases, phosphatases and other molecules could complicate the effects of tyrosine kinases and phosphatases. For instance, PKA and PKC crosstalk apparently complicates the regulation of hERG [59].

Both hERG and hEAG1 have several strong consensus sites for tyrosine phosphorylation [60]. It was recently shown that regulation of hEAG1 by the EGFR kinase involved three tyrosine sites (Tyr90, Tyr344, Tyr485) [25]. We do not know which site(s) in hERG were phosphorylated by Src and affected by the Src inhibitors. Future mutation analysis will be needed to determine whether other consensus sites for tyrosine phosphorylation (outside the ITIM) are involved in regulating hERG. We had hoped to observe more informative biophysical changes in the currents, given that some of the sites are in domains previously found to regulate the voltage dependence of activation, closing and inactivation. Our attempt to test whether our preferred candidate within the ITIM (Y827) is necessary for current regulation was not successful because there was no current in cells transfected with the hERG ITIM deletion mutant (amino acids 821–835), despite the presence of abundant ΔITIM-hERG protein. A trafficking defect was previously ruled out in the failure to express current for the cNBD deletion mutant (amino acids 815–825) [61]. In addition, our ΔITIM mutant retains the putative ER retention signal (R-X-R) at amino acids 1005-1007, which must be masked in order for hERG channels to be expressed in the surface membrane [62]. Several naturally occurring mutations exist in the cNBD of hERG, close to, but not within the ITIM. Mutation analysis within and/or near the cNBD has produced inconsistent results. Some missense mutations and truncations result in defective trafficking [63]–[69], while others show normal trafficking with impaired hERG channel function [61], [63], [68].

### Broader implications

There has been considerable structure-*versus*-function analysis of hERG and hEAG1 channels, especially concerning gating, but very little is known about roles of the conserved cyclic nucleotide binding domain (cNBD). Our work provides evidence that the ‘immuno-receptor tyrosine inhibitory motif’ (ITIM), which is within the cNBD, contributes to a multi-molecular complex, one role of which is to activate SHP-1 tyrosine phosphatase. This could allow SHP-1 to de-phosphorylate nearby target proteins including, but not necessarily limited to, the channel itself. Trans-activation of SHP-1 by EAG-family K^+^ channels and reciprocal regulation of the channels by Src and SHP-1 could be important for cell signaling that controls survival, proliferation, differentiation or other functions of many cell types. ERG1 and EAG1 channels are expressed in numerous cells and tissues in which signaling through tyrosine phosphorylation is important. Src is nearly ubiquitous and appears to regulate normal and pathological functions in cardiac, endothelial and vascular smooth muscle cells [18], [19], [70]–[72], which also express ERG1 [10], [73]. ERG1 channels in neurons and neuronal cell lines can modulate the membrane potential and excitability [74], [75], secretion [76] and neuritogenesis [77]–[78]. EAG1 is expressed most prominently in the brain and peripheral ganglia [1]–[3], and in myoblasts, its expression is associated with differentiation [4]. hEAG1 and hERG can also contribute to cell pathology. We provided the first evidence of selective up-regulation of hERG expression and function in primary leukemic cells and several hematopoietic cell lines [12], and the present results might be especially relevant to cancer. Both channels regulate proliferation and survival of the numerous cancer cell types in which they are aberrantly expressed [5]–[7], [11]–[14]. Tyrosine phosphorylation levels play important roles in many cancer cells, and an imbalance can lead to malignancies, as well as autoimmune diseases and inflammatory disorders [20]–[22]. SHP-1 expression is variable; it is dramatically decreased or even undetectable in lymphomas and most leukemia cells lines [79]–[82] but is increased in breast and ovarian cancer cell lines [83]–[85]. Changes in expression of this tyrosine phosphatase are expected to skew the balance of hERG and hEAG1 tyrosine phosphorylation. This might help explain why some cancer treatments using tyrosine kinase inhibitors show resistance to the drug over time.
